# Mental Health and Quality of Life in Glaucoma Patients: Insights From a Comparative Study

**DOI:** 10.7759/cureus.79241

**Published:** 2025-02-18

**Authors:** João Alves Ambrósio, Catarina Pestana Aguiar, Pedro Cardoso Teixeira, João Chibante Pedro, Jeniffer Jesus

**Affiliations:** 1 Ophthalmology Department, Unidade Local de Saúde de Entre Douro e Vouga, Santa Maria da Feira, PRT

**Keywords:** glaucoma, mental health, ophthalmology, physical and psychological well-being, psychosocial functioning, quality of life

## Abstract

Introduction

Glaucoma, a chronic and progressive optic neuropathy, is a leading cause of irreversible blindness, affecting both visual function and quality of life (QoL). The disease’s progression, alongside the fear of blindness, contributes to anxiety, depression, and reduced life satisfaction. This study aimed to evaluate the impact of glaucoma on mental health and QoL and to examine the relationship between glaucoma severity and psychological and functional factors.

Methods

A cross-sectional study was conducted involving 102 patients with glaucoma and 82 age-matched controls without significant ocular disease. All participants completed the 12-Item Short-Form Health Survey version 2 (SF-12v2) to assess general health and the Glaucoma QoL Questionnaire (Glau-QoL-17) for disease-specific QoL. Additional demographic and clinical data, such as education level, household composition, driving status, type of glaucoma, best-corrected visual acuity, number of antiglaucoma medications, static automated perimetry results, psychiatric comorbidities, use of psychiatric medications, and disease-related anxiety were also recorded.

Results

Glaucoma patients had a mean age of 72.3 ± 7.6 years, with 52.0% (n=53) being female, compared to a mean age of 70.1 ± 18.2 years in the control group, with a female population of 51.2% (n=49). Glaucoma patients scored significantly lower in general health compared to controls (Physical component score: 41.3 ± 11.3 vs. 49.7 ± 8.3* (p* < 0.001); Mental component score: 41.8 ± 10.8 vs. 51.0 ± 9.2* (p* < 0.001), affecting physical functioning, vitality, social functioning, and mental health. Disease-specific QoL was also worse in glaucoma patients (Glau-QoL-17 total score: 44 vs. 58, *p *< 0.001), particularly in anxiety, self-image, daily life activities, psychological state, constraints, and self-care. Psychiatric comorbidities were more frequent in glaucoma patients (46.1% vs. 22%*) (p* < 0.001), with 64.7% (n=66) reporting disease-related anxiety. Significant correlations were found between visual field loss severity and both physical (*p *= 0.028) and emotional (*p* = 0.035) role functioning, as well as general health perception (*p *= 0.015).

Conclusion

Glaucoma significantly impacts both general and disease-specific QoL, with more severe visual field impairment associated with poorer physical health outcomes. The high prevalence of psychiatric comorbidities and increased use of psychiatric medications further illustrate the psychological burden of glaucoma. These findings highlight the need for holistic care approaches that address both the physical and mental health aspects of glaucoma management.

## Introduction

Glaucoma is a chronic and progressive optic neuropathy and remains one of the leading causes of irreversible blindness worldwide [1]. It is characterized by the gradual loss of retinal ganglion cells, leading to progressive visual field impairment. This deterioration of vision can significantly hinder patients' ability to perform daily activities, such as driving and reading, and can reduce overall independence, especially as the disease advances [2]. As glaucoma is a lifelong condition requiring ongoing management, its impact extends beyond visual function, posing serious implications for mental health and quality of life (QoL) [2,3].

The psychological burden associated with glaucoma is multifaceted. Progressive vision loss, compounded by the chronic nature of the disease and the fear of impending blindness, may lead to increased levels of anxiety and depression [4,5]. Additionally, limitations in performing daily activities and the need for continuous medication can contribute to reduced life satisfaction and diminished psychological well-being [6]. Previous studies have indicated that glaucoma patients often report worse outcomes in terms of mental health compared to those without the condition [3,4,6].

While the clinical evaluation focused on intraocular pressure (IOP) control, visual field assessments, and optic nerve health is crucial for disease management, these assessments often overlook the patient’s subjective experience, including how they psychologically and emotionally cope with the disease, their adherence to treatment regimens, and the overall impact of glaucoma on their QoL. The European Glaucoma Society Guidelines highlight the importance of tailoring treatment strategies, including determining the target IOP, medication intensity, and frequency, based on the patient's individual QoL and ability to tolerate the prescribed treatment plan [7].

Despite being inherently subjective, QoL can be measured using validated instruments designed to objectively capture patients' experiences. Among the most used tools is the 12-Item Short-Form Health Survey version 2 (SF-12v2) [8], which evaluates both physical and mental well-being. This instrument covers several key domains, including physical functioning, role limitations due to physical health, vitality, social functioning, and mental health.

In addition to general health surveys, disease-specific tools have been developed to better assess the impact of glaucoma on QoL. One such instrument is the Glau-QoL-36 [9]. Originally developed in French, this questionnaire was designed to measure the impact of chronic open-angle glaucoma and its treatment on a patient’s QoL, capturing key areas such as treatment burden, daily life limitations, and emotional well-being. The Glau-QoL-36 has since been refined and shortened to the Glau-QoL-17 [10], which maintains the core dimensions of the original while being more practical for clinical and research settings. The Glau-QoL-17 is self-administered and measures seven components of QoL: anxiety, self-image, psychological state, daily life activities, driving, functional limitations, and control over the disease. This tool has recently been translated and validated in Portuguese, enabling its application in Portuguese-speaking populations [11].

Understanding and measuring QoL in glaucoma patients serves multiple purposes. First, it helps healthcare professionals educate patients about disease progression, reinforcing the importance of consistent therapy adherence, even when symptoms are absent. Many patients struggle with adherence due to glaucoma’s asymptomatic early stages, which can lead to a false sense of security. By showing how therapy can slow progression and improve QoL, clinicians can motivate patients to remain committed to their treatment. Second, incorporating QoL assessments into routine care strengthens the doctor-patient relationship. When clinicians consider patient-reported experiences, they foster empathy and trust, which can enhance treatment adherence.

This study aimed to assess the impact of glaucoma on mental health and QoL by evaluating general and glaucoma-specific QoL measures. Furthermore, we sought to explore the relationship between glaucoma severity, as measured by visual field impairment, and various psychological and functional factors, including anxiety, depression, and daily life limitations, in glaucoma patients compared to controls.

## Materials and methods

Study design and population

This was a cross-sectional study conducted at Unidade Local de Saúde Entre Douro e Vouga, Santa Maria da Feira, Portugal, involving 184 patients, 102 with a confirmed diagnosis of glaucoma and 82 age-matched controls without glaucoma. The study was approved by the Comissão de Ética para a Saúde da Unidade Local de Saúde de Entre Douro e Vouga (approval number: 13_2024). This study was conducted in accordance with the tenets of the Declaration of Helsinki.

Patients were recruited from the glaucoma outpatient clinic, while controls were drawn from a community population without significant ocular disease. Inclusion criteria for glaucoma patients included a diagnosis of primary open-angle glaucoma (POAG), pigmentary glaucoma, or pseudoexfoliation (PEX) glaucoma for more than six months, confirmed through clinical examination and visual field testing. Patients with dementia, psychiatric or neurological conditions who were not able to answer the QoL questionnaires and patients with other ophthalmological conditions that could influence visual acuity were excluded.

Data collection instruments

All participants completed two standardized questionnaires: (i) SF-12v2, which includes questions covering general health, vitality, bodily pain, and social functioning, along with two questions each from the role-emotional, mental health, physical functioning, and role-physical subscales [8]. The Physical Component Score and Mental Component Score provide a comprehensive view of overall health and mental well-being. Higher scores indicate better QoL; (ii) Glau-QoL-17, which is a disease-specific tool containing 17 items across seven dimensions: anxiety (three items), self-image (two items), psychological (two items), daily life (four items), driving (two items), constraints (two items), and self-care (two items). Higher scores on the Glau-QoL-17 indicate a better QoL.

Additional demographic and clinical data

Additional demographic data, including education level, household composition (living alone vs. accompanied), and driving status, were collected through self-reporting. Clinical data collected from glaucoma patients included the type of glaucoma, best-corrected visual acuity (BCVA) of the worst eye, use and number of antiglaucoma medications, static automated perimetry results (mean deviation (MD) and pattern standard deviation [PSD]) of the most affected eye, presence of psychiatric comorbidities, use of psychiatric medications, and disease-related anxiety levels.

Visual field and glaucoma severity assessment

The severity of glaucoma was categorized based on visual field impairment as mild, moderate, or severe, according to the Hodapp-Parrish-Anderson classification [12]. Visual field testing 24-2 was performed using static automated perimetry (Humphrey Field Analyzer^TM^ II-745i; Carl Zeiss Meditec AG, Jena, Germany), and the MD score was used as a marker of disease severity.

Statistical analysis

Descriptive statistics were used to summarize demographic and clinical characteristics. Independent sample t-tests and chi-square tests were employed to compare continuous and categorical variables, respectively, between glaucoma patients and controls. For group comparisons, a one-way analysis of covariance (ANCOVA) was used to adjust for confounding factors such as age, sex, education level, driving status, and household composition. Correlation analyses were conducted to assess the relationship between visual field impairment and QoL outcomes. A p-value <0.05 was considered statistically significant for all analyses. Data were analyzed using IBM SPSS Statistics for Windows, Version 29.0 (2022; IBM Corp., Armonk, New York, United States). SF-12 composite scores were generated according to standard algorithms [13]. For the Glau-QoL-17, no item weighting was applied, and the score was the sum of the items for each dimension​ [10]. 

## Results

Demographic and clinical characteristics

The demographic characteristics of the glaucoma and control group are summarized in Table 1. The study included 102 patients with glaucoma and 82 age-matched controls. The mean age of the glaucoma patients was 72.3 ± 7.6 years, compared to 70.1 ± 18.2 years in the control group, with no significant difference in age between the groups (*p* = 0.312). The gender distribution was also comparable, with 52.0% of the glaucoma group and 51.2% of the controls being female (*p* = 0.920). Educational level differed significantly between the groups (*p* < 0.001), with a higher proportion of controls achieving higher levels of education. A similar proportion of participants in both groups reported driving (52.0% of glaucoma patients vs. 52.4% of controls, *p* = 0.949) and living with others (84.3% of glaucoma patients vs. 89.0% of controls, *p* = 0.354).

**Table 1:** Participant demographics p-values from ^a^Qui-square Test, ^b^Independent samples t Test, ^c^Fisher Exact Test

	Glaucoma patients (n = 102)	Controls (n = 82)	p-value
Sex, n (%)			0.920^a^
Female	53 (52.0)	42 (51.2)	
Male	49 (48.0)	40 (48.8)	
Age (years), mean±SD	72.3 ± 7.6	70.1 ± 18.2	0.312^b^
Education level, n (%)			<0.001^c^
Illiterate	1 (1.0)	0	
Literate (no formal education)	14 (7.6)	1 (1.2)	
Primary Education (Year 4)	56 (54.9)	39 (47.6)	
Primary Education (Year 6)	4 (2.2)	4 (4.9)	
Lower Secondary Education (Years 7–9)	3 (2.9)	2 (2.4)	
Upper Secondary Education (Year 10)	17 (16.7)	8 (9.8)	
Upper Secondary Education (Year 12 / A-Levels)	5 (4.9)	12 (14.6)	
Higher Education (University or equivalent)	2 (2.0)	16 (19.5)	
Driving, n (%)	53 (52.0)	43 (52.4)	0.949^a^
Household status, n (%)			0.354^a^
Alone	16 (15.7)	9 (11.0)	
Living with others	86 (84.3)	73 (89.0)	

Among the glaucoma patients, 37.3% had POAG, 60.8% had PEX glaucoma, and 2.0% had pigmentary glaucoma. Bilateral disease was present in 86.3% of glaucoma patients. Regarding treatment, 52.9% of patients were subjected to glaucoma surgery, and 9.8%, 48.0%, 39.2%, and 2.9% of patients used none, one, two, or three pressure-lowering eye drop bottles, respectively. Most glaucoma patients were pseudophakic (66.7%). The mean BCVA was 0.1 (0.2) logMAR. Visual field measures were missing in 33 patients, as static automated perimetry was not performed or was substituted by the Goldmann visual field. Based on the MD score, the severity of glaucoma was classified as mild in 26.1%, moderate in 17.4%, and severe in 56.5% of patients [12]. The mean PSD was 8.16 ± 3.60.

Mental health and QoL outcomes

The QoL measures are summarized in Table 2. Psychiatric comorbidities were significantly more prevalent in the glaucoma group (46.1% vs. 22.0%, *p* < 0.001), and the use of psychiatric medications was also higher (46.1% vs. 20.5%, *p* = 0.005). Anxiety related to the disease was reported by 64.7% of glaucoma patients.

**Table 2:** Mental health and quality of life outcomes P-values from ^a^Qui-square Test, ^b^One-Way ANCOVA, ^c^Independent Sample T Test, ^d^Mann-Whitney U *Adjusted to age, sex, education level, driving, and household. IQR: interquartile range

	Glaucoma patients (n = 102)	Controls (n = 82)	*p*-value	Adjusted p*
General mental health, n (%)				
Psychiatric pathology	47 (46.1)	18 (22)	<0.001^a^	0.002^b^
Psychiatric medication	47 (46.1)	8 (20.5)	0.005^a^	0.008^b^
Disease-related anxiety	66 (64.7)	N/A	N/A	N/A
SF-12, mean±SD				
General Physical Score	41.3 ± 11.3	49.7 ± 8.3	<0.001^c^	<0.001^b^
General Mental Score	41.8 ± 10.8	51.0 ± 9.2	<0.001^c^	<0.001^b^
Physical functioning	43.1±13.5	51.8 ± 8.0	<0.001^c^	<0.001^b^
Role – physical	41.1 ± 11.5	49.7 ± 8.4	<0.001^c^	<0.001^b^
Bodily pain	44.3 ± 11.1	49.2±12.0	0.005^c^	0.051^b^
General health	35.2 ± 13.0	47.6±10.3	<0.001^c^	<0.001^b^
Vitality	44.0±11.4	54.3±9.3	<0.001^c^	<0.001^b^
Social functioning	36.3±11.7	48.9±9.2	<0.001^c^	<0.001^b^
Role – emotional	38.9±12.9	48.4±8.0	<0.001^c^	<0.001^b^
Mental health	46.5±10.9	52.6±10.4	<0.001^c^	<0.001^b^
Glau-QoL-17				
Total, n (%)	44 (19)	58 (7)	<0.001^d^	<0.001^b^
Anxiety, mean±SD	6.6 ± 3.3	10.1 ±1.6	<0.001^c^	<0.001^b^
Self-image, mean±SD	2.6 ±1.3	4.1 ±1.4	<0.001^c^	<0.001^b^
Psychological, mean±SD	4.7± 2.4	6.4±1.7	<0.001^c^	<0.001^b^
Daily life, median (IQR)	12 (7)	14 (2)	0.002^d^	<0.001^b^
Driving, median (IQR)	8 (2)	8 (1)	0.077^d^	0.057^b^
Constraints, median (IQR)	6 (3)	8 (0)	<0.001^d^	<0.001^b^
Care, median (IQR)	4 (1)	8 (0)	<0.001^d^	<0.001^b^

Glaucoma patients reported significantly lower scores on the SF-12v2 compared to controls. The Physical Component Score was 41.3 ± 11.3 in glaucoma patients, significantly lower than the 49.7 ± 8.3 in controls (*p* < 0.001). The Mental Component Score was also reduced in glaucoma patients (41.8 ± 10.8 vs. 51.0 ± 9.2, *p* < 0.001). Glaucoma patients scored lower across several SF-12 subdomains, including physical functioning (*p* < 0.001), role-physical (*p* < 0.001), vitality (*p* < 0.001), social functioning (*p* < 0.001), and mental health (*p* < 0.001). Similarly, they had significantly lower scores on the Glau-QoL-17 questionnaire, particularly in areas related to anxiety (*p* < 0.001), self-image (*p* < 0.001), psychological state (*p* < 0.001), daily life (*p* < 0.001), constraints (*p* < 0.001), and self-care (*p* < 0.001).

Correlations with visual field loss

The SF-12v2 results by glaucoma severity are summarized in Table 3. There is a significant association between visual field impairment and both physical (*p* = 0.028) and emotional (p = 0.035) role functioning. More severe visual field loss was associated with poorer general health perceptions (*p* = 0.015), lower scores in physical functioning (*p* = 0.004), and a lower General Physical Score (*p* = 0.018). However, no significant differences were observed across glaucoma severity levels in terms of anxiety, self-image, psychological state, daily life, constraints, and self-care in the disease-specific QoL questionnaire (*p* > 0.05).

**Table 3:** SF-12v2 scores by glaucoma severity P-values from ^a^One-Way ANOVA SF-12v2: 12-Item Short-Form Health Survey version 2 [8]

	Mild (n = 18), mean ± SD	Moderate (n = 12), mean ± SD	Severe (n = 39), mean ± SD	*p*-value
General Physical Score	48.2 ± 9.5	37.6 ± 9.9	40.2 ± 11.9	0.018^a^
General Mental Score	44.7 ± 13.2	42.8 ± 9.9	40.9 ± 9.3	0.458^a^
Physical functioning	51.7 ± 10.3	36.4 ± 12.9	41.9 ± 13.4	0.004^a^
Role – physical	47.2 ± 10.6	37.6 ± 10.0	39.6 ± 11.3	0.028^a^
Bodily pain	46.7 ± 13.7	43.9 ± 12.5	44.9 ± 10.3	0.789^a^
General health	43.3 ± 12.9	34.1 ± 10.1	33.1 ± 12.3	0.015^a^
Vitality	47.7 ± 11.9	45.2 ± 12.9	42.8 ± 9.7	0.289^a^
Social functioning	40.3 ± 10.5	38.0 ± 12.8	34.8 ± 10.9	0.216^a^
Role – emotional	45.2 ± 12.4	33.2 ± 12.9	38.4 ± 12.3	0.035^a^
Mental health	48.9 ± 12.9	47.8 ± 8.7	45.5 ± 10.4	0.501^a^

## Discussion

This study highlights the substantial impact of glaucoma on both mental health and QoL, corroborating previous findings that glaucoma patients experience a heightened psychological burden [3-6]. Our results demonstrate that glaucoma patients report significantly lower scores on both general and disease-specific QoL measures compared to age-matched controls. These lower scores persist even after adjusting for confounding factors such as age, sex, education level, driving status, and household composition, underscoring the role glaucoma plays as a psychological disruptor, independent of demographic variables.

The high prevalence of psychiatric comorbidities among glaucoma patients, 46.1% in our study, aligns with earlier research indicating elevated rates of anxiety and depression in this population [4-6]. Furthermore, the use of psychiatric medications among 46.1% of glaucoma patients, not all of whom had a diagnosed psychiatric disorder, emphasizes the need for integrated management approaches that address both ocular and mental health. Of particular concern is that 64.7% of glaucoma patients in our study reported disease-related anxiety, underscoring the need for ophthalmologists to actively address these psychological concerns rather than relying solely on mental health specialists. To achieve this, ophthalmologists can play a crucial role in identifying at-risk patients by incorporating brief mental health screenings into routine glaucoma assessments to help detect symptoms of anxiety and depression. Patient education on the psychological impact of glaucoma, reassurance regarding disease progression, and clear referral pathways for psychological support should be integral to clinical care. Additionally, fostering multidisciplinary collaboration between ophthalmologists and mental health professionals may further enhance patient outcomes by ensuring timely interventions for those experiencing significant distress.

In the Glau-QoL-17 questionnaire, glaucoma patients reported higher levels of anxiety than controls, likely due to concerns about missing their target IOP, fear of losing vision, or the potential need for surgery. Self-image was also notably worse in glaucoma patients, many of whom avoided discussing their ocular issues and reported feeling prematurely aged. Psychological state scores were lower as well, with glaucoma patients feeling more discouraged and vulnerable. Daily life activities were more affected in the glaucoma group, with difficulties such as reading labels, watching television, recognizing familiar faces, and taking longer to complete routine tasks. A significant finding was that almost half of the glaucoma patients disagreed or strongly disagreed with the statement, "Nowadays, I have enough information about my vision problems", one of the individual questions from the Glau-QoL-17 questionnaire. This indicates a communication gap between ophthalmologists and patients and suggests that improved patient education could positively impact mental health and QoL. Research has shown that patients with less information about their disease exhibit higher anxiety and depression scores, highlighting the critical role of patient education in reducing psychological distress [14].

Our study also found a significant association between glaucoma severity and reduced physical functioning and emotional well-being, suggesting that progressive vision loss contributes directly to physical limitations and emotional distress. As the disease advances, patients may face increasing difficulties in performing daily activities, which can exacerbate anxiety and depression, further reducing life satisfaction [15]. Interestingly, while glaucoma severity subgroups showed significant differences in the physical components of the SF-12v2, such differences were not observed in the mental health components. This suggests that the mental burden of the disease is present from the moment of diagnosis and does not necessarily worsen with disease progression [16]. Additionally, in the Glau-QoL-17 questionnaire, no significant differences were observed across glaucoma severity levels for anxiety, self-image, psychological state, daily life, constraints, or self-care. This finding may indicate that distress stems from the diagnosis itself, the uncertain prognosis, and the need for lifelong treatment, rather than the degree of visual field loss alone.

Our findings underscore the importance of considering the psychological aspects of glaucoma management, particularly for patients with more advanced visual field loss. Addressing the mental health needs of glaucoma patients is crucial for improving overall QoL. Incorporating mental health screening and counseling into routine glaucoma care could help alleviate the psychological burden associated with the disease. Additionally, improving patient education about glaucoma, its progression, and available treatment options may reduce anxiety and empower patients to better manage their condition [7,16,17].

Several limitations should be acknowledged. The reliance on self-reported medical conditions may introduce recall bias and inaccuracies. Furthermore, the statistically significant difference in educational levels between glaucoma and control groups, where controls had a higher proportion of participants with advanced education, may reflect selection bias, as more educated individuals may be more willing to participate in research and complete questionnaires. Additionally, the fact that driving status was similar between the two groups is important, as the inability to drive could act as a confounding factor that would affect QoL outcomes [18]. Similarly, household composition, whether living alone or with others, could influence mental health, as living alone is often associated with reduced mental well-being [19]. Another limitation was the exclusion of 33 glaucoma patients from the severity analysis due to missing visual field data. The exclusion of these patients, likely with more advanced disease and possibly unable to undergo computerized static perimetry, may have potentially underestimated the differences in physical scores between groups [2,3].

## Conclusions

This study demonstrates that glaucoma significantly impacts both general and disease-specific QoL, with more severe visual field impairment linked to poorer physical health outcomes. The high prevalence of psychiatric comorbidities and the frequent use of psychiatric medications among glaucoma patients highlight the considerable psychological burden associated with the disease. These findings emphasize the need for comprehensive, integrated care approaches that address both the visual and psychological aspects of glaucoma management.
